# Supplementary material for: A petascale automated imaging pipeline for mapping neuronal circuits with high-throughput transmission electron microscopy
Source: Nat Commun. 2020 Oct 2;11:4949. doi: 10.1038/s41467-020-18659-3 (PMC7532165; doi:10.1038/s41467-020-18659-3)
Supplement: Supplementary file 1 — Supplementary Information [file 41467_2020_18659_MOESM1_ESM.pdf]

SUPPLEMENTARY METHODS

Multi-System Monitoring (MSM)

Traditionally most electron microscopy projects are centered on one imaging machine whereas the piTEAM pipeline utilizes multiple machines running in parallel. The need to quickly diagnose failures in a multi-system environment requires a robust method of troubleshooting as well as active preventative maintenance. In order to satisfy these requirements, a monitoring system (MSM) for piTEAM and the laboratory environment was implemented.

MSM tracks three top level systems (Supplementary Figure 5) that each contain an assembly of sub-systems: Facilities, Environment, and Equipment. Facility related sub-systems include the status of the pressure, flow, and temperature of chilled water circulating within each system. The environmental sub-systems include the status of laboratory conditions such as room temperature and humidity. Equipment sub-systems include the status of each autoTEM including filament life, vacuum pressure, and beam current. Image acquisition parameters are also tracked including focus scores, current machine activity, CPU, and memory use, and local disk space availability.

Success rates of sample preparation and major sources of imaging downtime:

No specimen was lost during en-bloc staining. The loss-rate of section is about 0.2% during ATUM sectioning and 0.1% during imaging due to non-manufacturing errors. The computational steps of stitching, alignment and segmentation did not cause section loss. The major sources of downtime (35%) vary across different autoTEMs but are roughly divided into the followings:

1. Barcode reading failures during sample translation: 15%
2. Microscope downtime such as HT failure, vacuum failure: 15%

3. Filament change including alignment: 5%

4. Camera calibration is usually done before the imaging of a new tape starts and is also checked on a monthly basis: 2 hours

5. Tape calibration time cost is minimal, each calibration takes a few minutes and is also done before the imaging of a new tape starts.

Manpower to install and run imaging pipeline

After a TEM system that matches the requirements for an autoTEM microscope is installed at a facility a team of three can accomplish the installation of the remaining components. Installation of microscope hardware can be accomplished by two individuals. The software installation can be done by a skilled software technician. Finally, an integration test should be performed by the end user(s) to validate the systems' readiness, often with the aid of the software personnel. Once operational the imaging pipeline has built-in automation and system control feedback to keep microscopes running by themselves 24/7. In general, it takes about 30% of an engineer's time during production to perform following tasks: error state recovery; QC, trouble-shooting scopes, tape and stage issues.

For the tissue preparation (histology and embedding) the manpower required is not specific for the tape collection or requires additional human resources compared with other sectioning approaches. The shape of the tissue block is specific to the tape method, but the manpower and time required is similar to others method and dependent on block thickness (for 1 millimeter it takes ~1 week/1 person to be done perfectly and to minimize risk of damage). Finally, it takes between 17-26 seconds per section when cutting.

SUPPLEMENTARY FIGURES

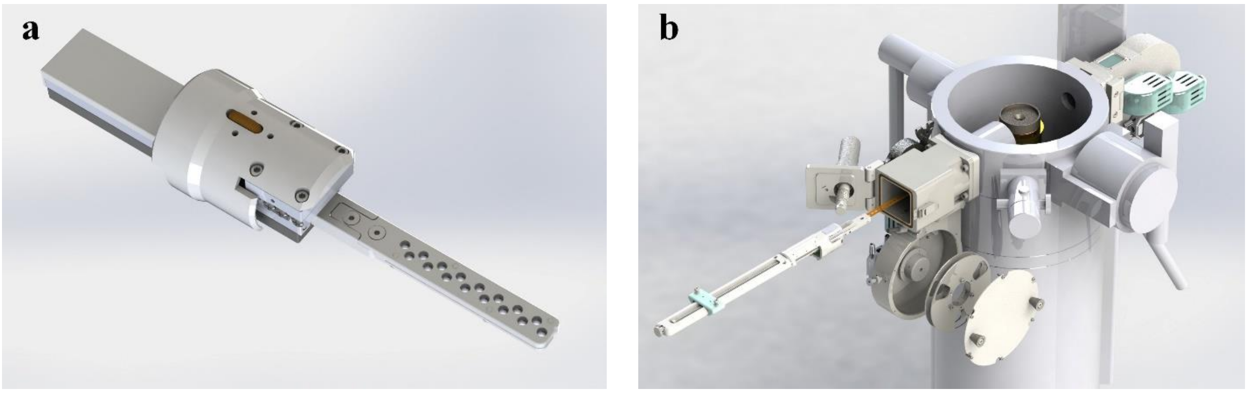

Supplementary Figure 1. Microscope stages a. 3D rendering of the GridStage Sprite used to image sections collected onto standard grids. Note the Grid Stick has 16 wells for holding standard TEM grids. b. 3D rendering of GridStage Reel and its reel-to-reel sample translation system.

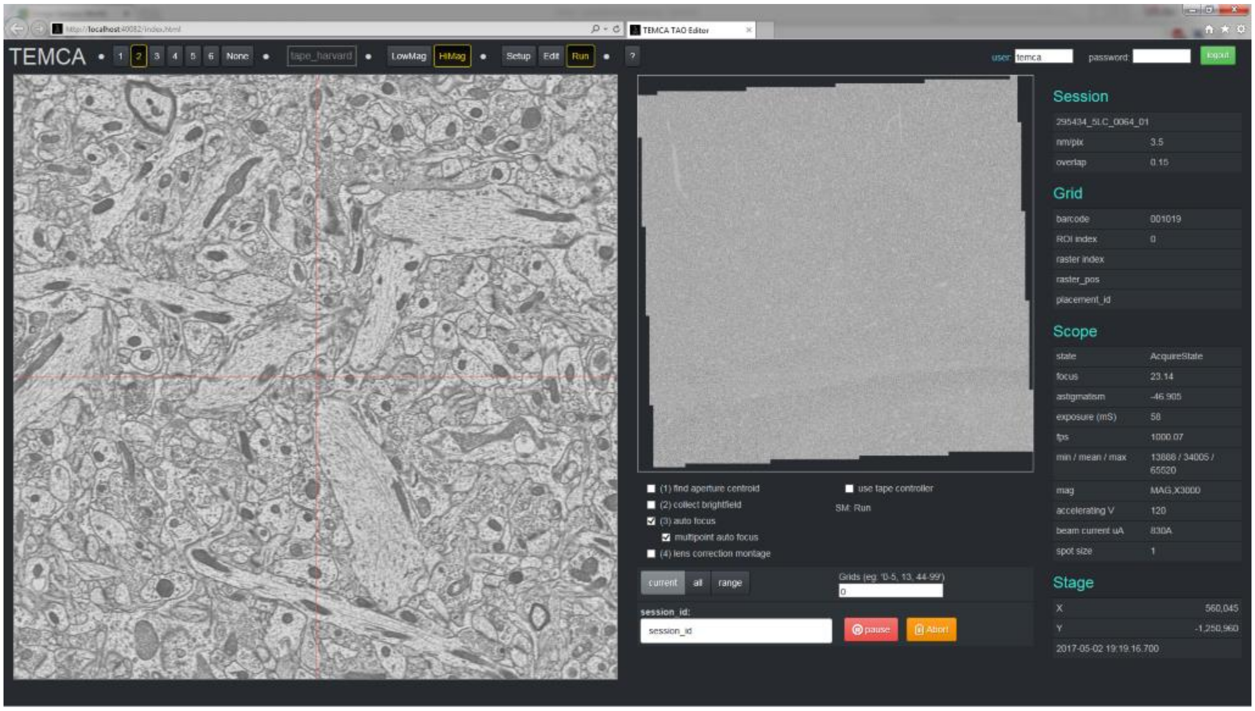

Supplementary Figure 2. Screen capture from pyTEM GUI live streaming. The left panel displays downsized high-res tiles during raster scanning. The right panel displays the montage and real-

time running parameters such as the section information, scope and stage status. pyTEM GUI uses responsive web design principles and operates at any screen resolution even on a mobile phone.

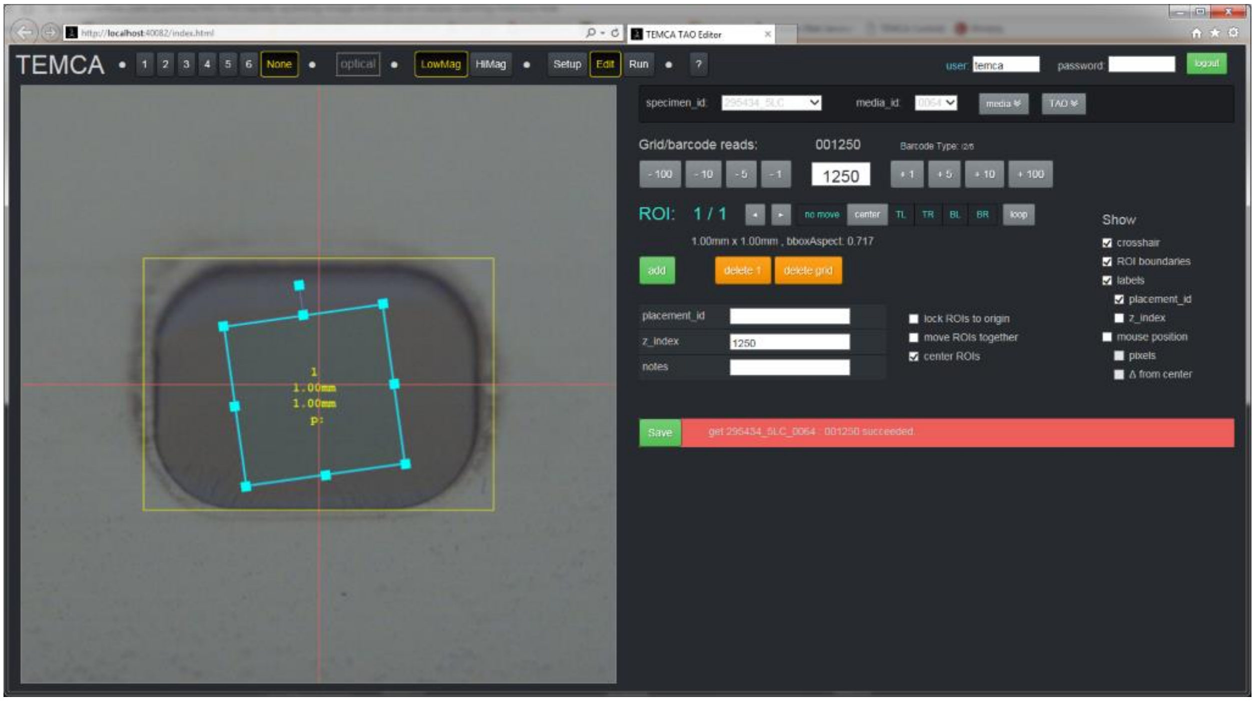

Supplementary Figure 3. User interface to define ROIs for high-fidelity raster imaging with pyTEM GUI.

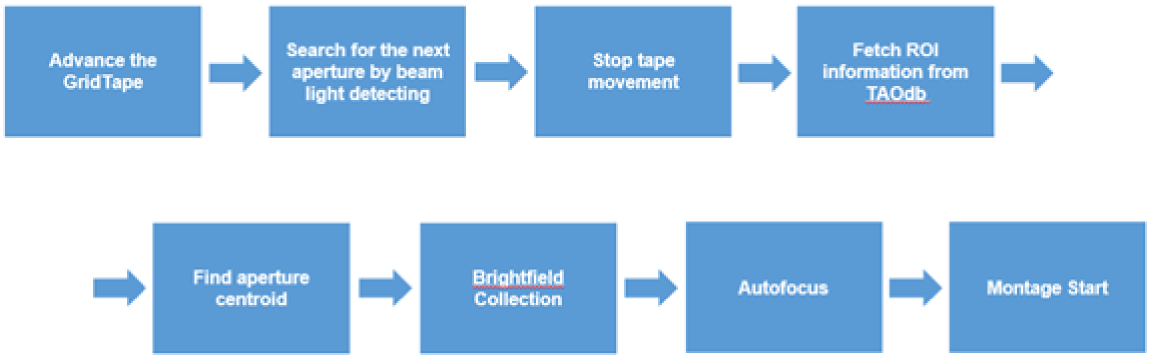

Supplementary Figure 4. Automated Flow for montage acquisition. The tape is advanced to the desired ROI. An ROI associated with the aperture is pulled from a cloud database. The centroid of the aperture is found via edge detection. A brightfield is collected and applied to all subsequent images linked with the ROI. Finally, an autofocus operation is performed to maximize image quality and then the montage raster scan is initiated.

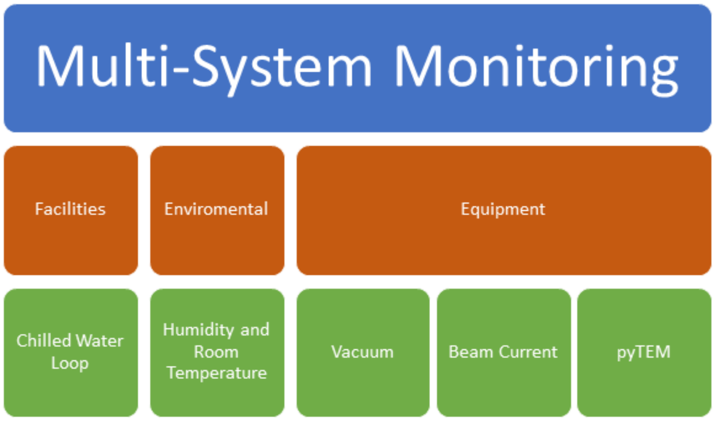

Supplementary Figure 5. Hierarchical schematic of the autoTEM Multi System Monitoring (MSM). Bottom up reporting of sub-components and sensors are integrated into a unified monitoring software system for easy diagnosing. All information is logged to a cloud database.

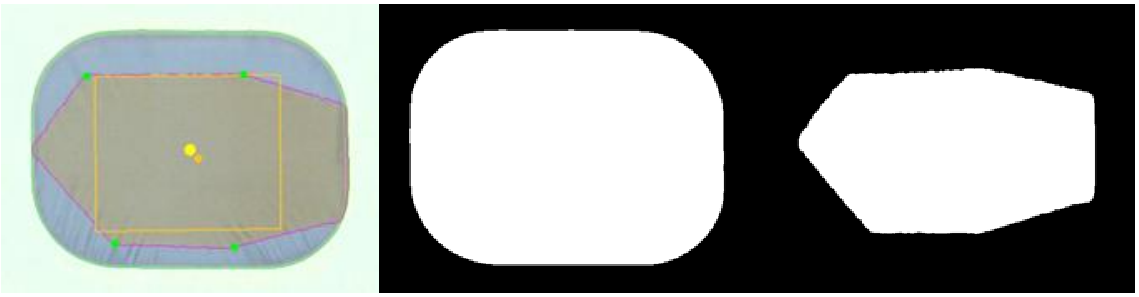

Supplementary Figure 6. Auto ROI definition from an optical aperture image (left). The aperture and tissue boundaries (middle and right) are automatically detected through machine vision, and an ROI is placed according to the distance offset and tissue compression scaling factor.

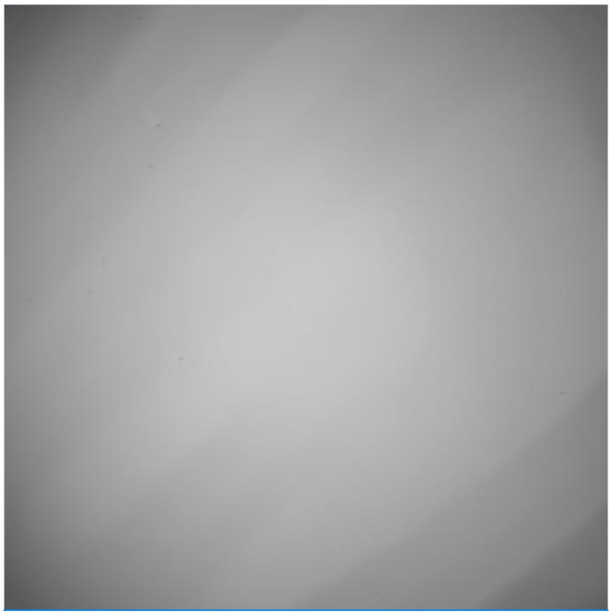

Supplementary Figure 7. Example brightfield collected from an autoTEM using 50Mpix camera.

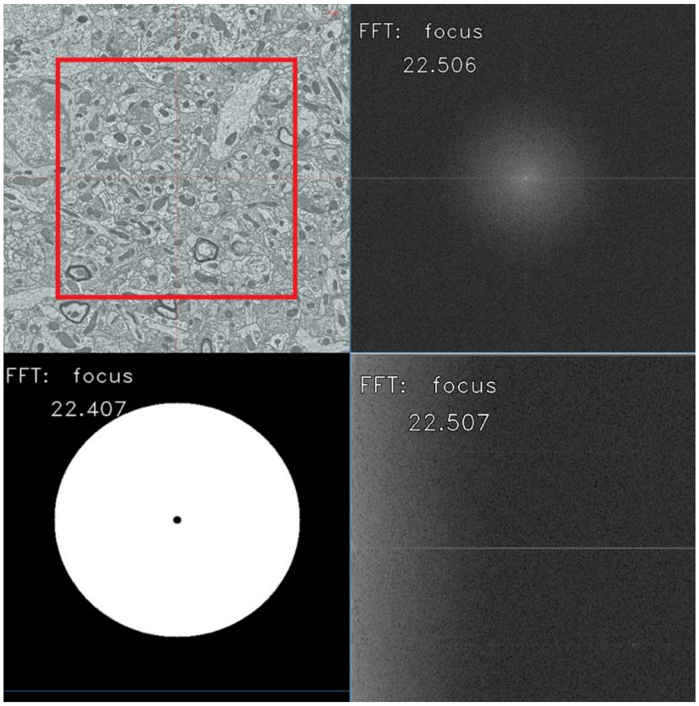

Supplementary Figure 8. Derivation of Autofocus algorithm. TL: focus is measured from center boxed 2048 × 2048 pixels out of a frame of 3840 × 3840 pixels. TR: DFT, BL: frequency range mask, BR: polar to rectangular conversion of DFT within mask.

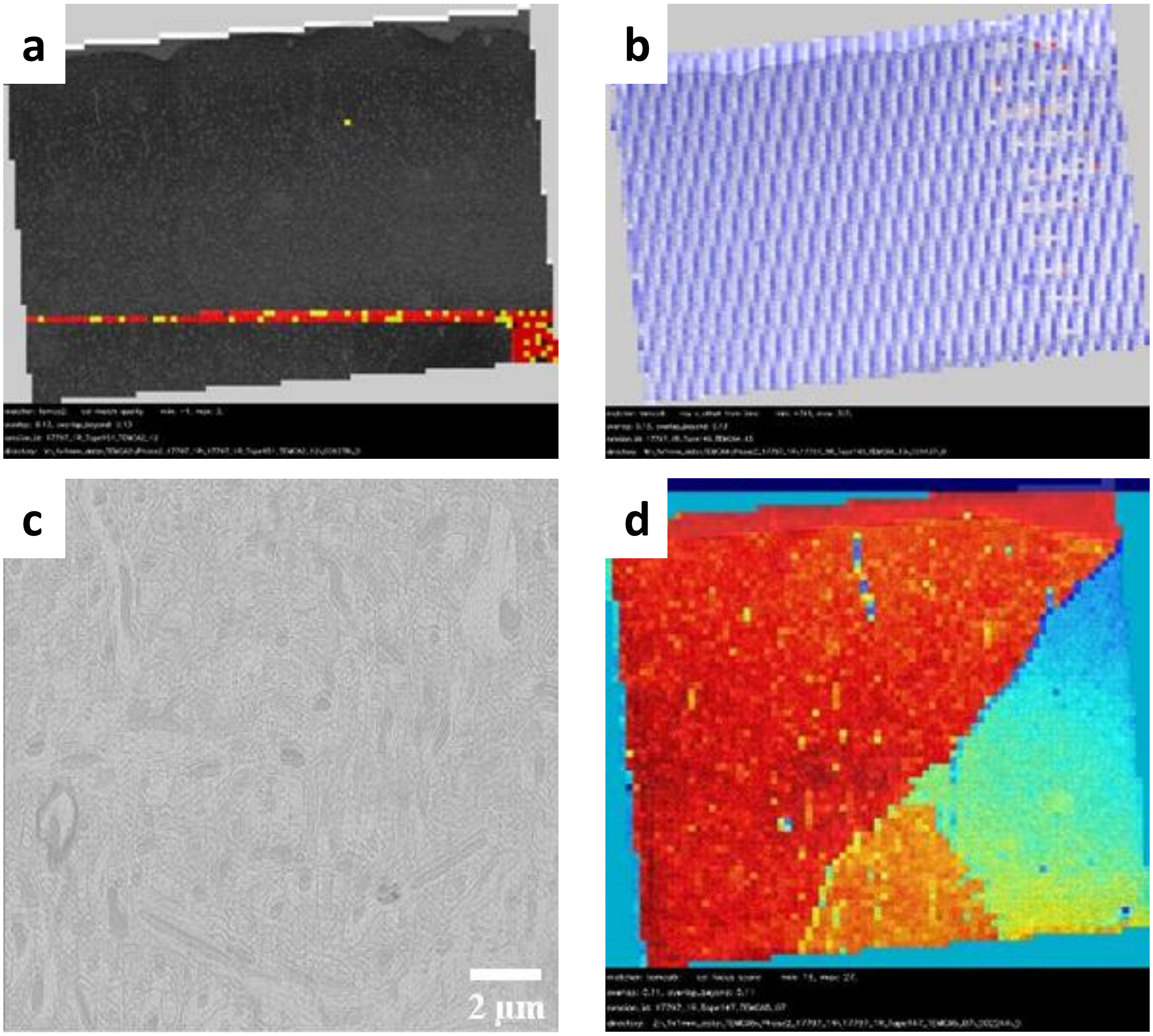

Supplementary Figure 9. Montage QC Failure Examples. a. Example of tape slippage, in which case the GridTape is not secured in-place on the stage and shifts the position during stage movement. Montage size: 1.2 mm × 0.85 mm. As a result, the montage quality map highlights misaligned rows and areas, or sometimes seen as partially imaged ROI. During 1 mm^3 imaging,

most of the reimaging needs came from tile overlap issue. Due to stage movement errors, there was a small fraction of tiles (< 1%) that did not have enough overlap to their neighboring tiles, usually along x-axis, and thus the image processing pipeline had difficulty finding point matchers within the overlap region (Supplementary Figure 10.b) to perform the stitching and alignment. b. Insufficient tile overlap seen as non-uniform pattern. Montage size: 1.2 mm × 0.85 mm. c. Random blurry tiles occurring because of charging, autofocus failure, or the stage not having enough settling time and thus a frame is captured by camera while stage is still moving. d. Gradient across the montage focus map where the right side is impacted by electron charging and tiles are slightly blurry. Montage size: 1 mm × 1 mm. Currently the top recurrent failure modes during imaging are intermittent beam intensity variation (at the end of filament life), intermittent focus deviation due to stage error or vibration. We used to encounter charging issues, but it has been pretty much eliminated after the GridStage modification. The barcode reading has also seen substantially improvement over the last two years through upgrades in reel-to-reel system as well as better barcode reading algorithm.

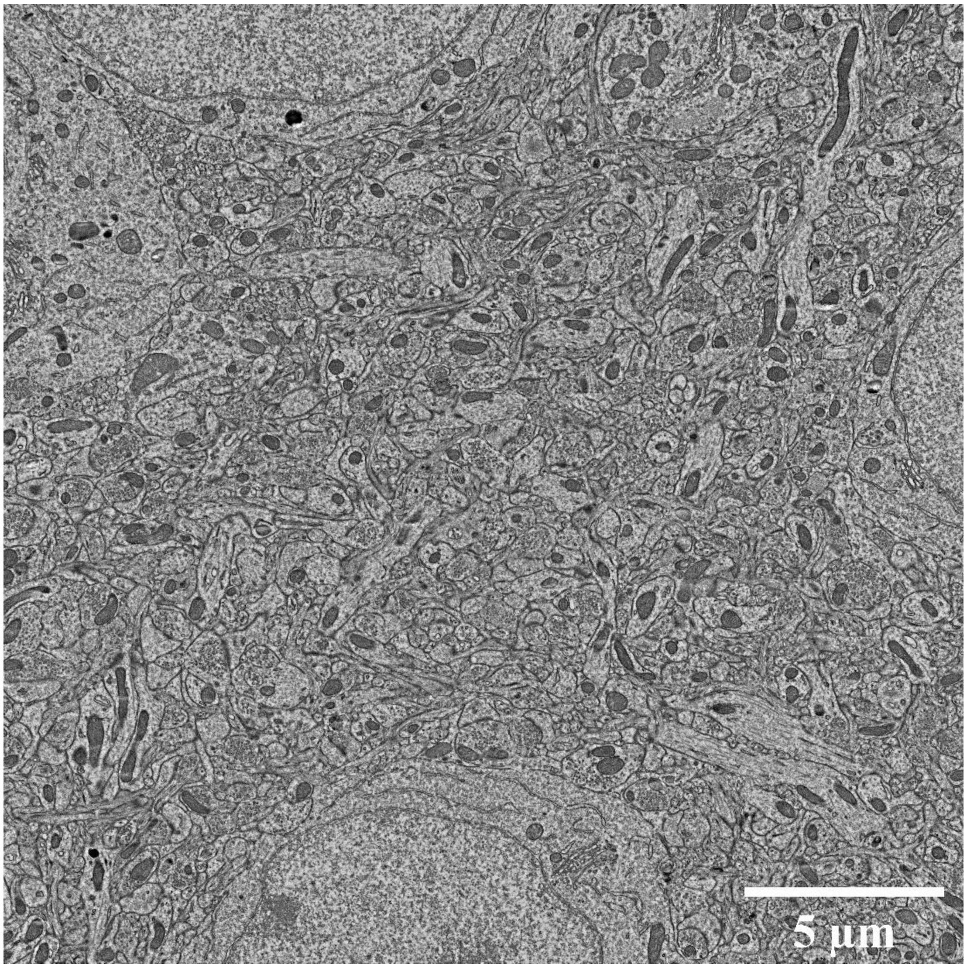

Supplementary Figure 10. High-res image collected by AMT lens assembly using a 50MP XIMEA camera sensor. The scope magnification is at 2500x. The pixel resolution is approximately 4nm/pixel.

SUPPLEMENTARY TABLE

Camera Model	XiB 50 Mpixel	> 100 Mpixel
Frame Size (pixels)	5408	10000
FOV (microns)	22	40
Tile Overlap (%)	9	5
Tiles w/overlap (per edge)	54	28
Total Tiles/Section	2916	784

Supplementary Table 1: Imaging metric comparison between current 50MP sensor vs future 100MP sensor (projected). The values are calculated using 4 nm per pixel in spatial resolution and a ROI size of 1 mm^2.
