# Supplementary material for: A petascale automated imaging pipeline for mapping neuronal circuits with high-throughput transmission electron microscopy
Source: Nat Commun. 2020 Oct 2;11:4949. doi: 10.1038/s41467-020-18659-3 (PMC7532165; doi:10.1038/s41467-020-18659-3)
Supplement: Supplementary file 3 — Description of Additional Supplementary Files [file 41467_2020_18659_MOESM3_ESM.pdf]

Description of Additional Supplementary Files

File Name: Supplementary Movie 1
Description: Software user interface, showing simultaneously real time acquisition over 5 microscopes. The left panel display individual frames, while the right panel shows imaging progression in individual microscopes over a mm^2 montage.

File Name: Supplementary Movie 2
Description: Montage of electron micrographs of layer 2/3 of mouse visual cortex.

File Name: Supplementary Movie 3
Description: Montage of electron micrographs of multiple layers human Medial Temporal Gyrus.

File Name: Supplementary Movie 4
Description: Individual electron micrographs from multiple layers of mouse visual cortex.
