# Supplementary material for: A petascale automated imaging pipeline for mapping neuronal circuits with high-throughput transmission electron microscopy
Source: Nat Commun. 2020 Oct 2;11:4949. doi: 10.1038/s41467-020-18659-3 (PMC7532165; doi:10.1038/s41467-020-18659-3)
Supplement: Supplementary file 8 — Reporting Summary [file 41467_2020_18659_MOESM8_ESM.pdf]

nature research

Corresponding author(s): Nuno da Costa

Last updated by author(s): Aug 17, 2020

Reporting Summary

Nature Research wishes to improve the reproducibility of the work that we publish. This form provides structure for consistency and transparency in reporting. For further information on Nature Research policies, see Authors & Referees and the Editorial Policy Checklist.

Statistics

For all statistical analyses, confirm that the following items are present in the figure legend, table legend, main text, or Methods section.

n/a	Confirmed	
☐	☒	The exact sample size (*n*) for each experimental group/condition, given as a discrete number and unit of measurement
☐	☒	A statement on whether measurements were taken from distinct samples or whether the same sample was measured repeatedly
☒	☐	The statistical test(s) used AND whether they are one- or two-sided<br>*Only common tests should be described solely by name; describe more complex techniques in the Methods section.*
☒	☐	A description of all covariates tested
☒	☐	A description of any assumptions or corrections, such as tests of normality and adjustment for multiple comparisons
☐	☒	A full description of the statistical parameters including central tendency (e.g. means) or other basic estimates (e.g. regression coefficient) AND variation (e.g. standard deviation) or associated estimates of uncertainty (e.g. confidence intervals)
☒	☐	For null hypothesis testing, the test statistic (e.g. *F*, *t*, *r*) with confidence intervals, effect sizes, degrees of freedom and *P* value noted<br>*Give P values as exact values whenever suitable.*
☒	☐	For Bayesian analysis, information on the choice of priors and Markov chain Monte Carlo settings
☒	☐	For hierarchical and complex designs, identification of the appropriate level for tests and full reporting of outcomes
☒	☐	Estimates of effect sizes (e.g. Cohen's *d*, Pearson's *r*), indicating how they were calculated

Our web collection on statistics for biologists contains articles on many of the points above.

Software and code

Policy information about availability of computer code

Data collection	Custom code: OpenCVGraph; pytemca; temca_gui; temca_db; temca_server; File storage system in Allen Institute<br>Open source: OpenCV 3.4; C++; Python 3.6; Cuda 10; Visual Studio 2017; Visual Studio Code 1.40<br>Commercial: GridCon 1.4.0 (Voxa); AWS
Data analysis	Open source: Any tiff/jpeg file image viewer; Visual Studio Code 1.40; Python 3.6; iPython Notebook; PyQt 5<br>Custom code: pytemca; temca_gui; tile_qc_meta;

For manuscripts utilizing custom algorithms or software that are central to the research but not yet described in published literature, software must be made available to editors/reviewers. We strongly encourage code deposition in a community repository (e.g. GitHub). See the Nature Research guidelines for submitting code & software for further information.

Data

Policy information about availability of data

All manuscripts must include a data availability statement. This statement should provide the following information, where applicable:

- Accession codes, unique identifiers, or web links for publicly available datasets
- A list of figures that have associated raw data
- A description of any restrictions on data availability

This manuscript is about the methodology to acquire the petabyte size neuroanatomical dataset with more than one hundred million images. The datasets were collected at a large-scale facility within Allen Institute. The authors have attached example image data in the supplementary videos. More electron microscopy image data that supports the findings of this work are available on www.microns-explorer.org and on https://github.com/AllenInstitute/piTEAM

Field-specific reporting

Please select the one below that is the best fit for your research. If you are not sure, read the appropriate sections before making your selection.

☒ Life sciences ☐ Behavioural & social sciences ☐ Ecological, evolutionary & environmental sciences

For a reference copy of the document with all sections, see nature.com/documents/nr-reporting-summary-flat.pdf

Life sciences study design

All studies must disclose on these points even when the disclosure is negative.

Sample size	Dataset 1: 1 mm^3 mouse visual cortex block (Slc-Cre/GCaMP6s, M, age P60): . 26500 sections (40nm) of 1mm^2 montages. Over 100 million high-resolution EM images. 2PB total data size; Dataset 2: pipeline test dataset. 1000 tissue sections of 1mm^2 montages (CamKII-tTA/tetO-GCaMP6s, M, age P49) ; Dataset 3: pipeline test dataset. 2500 tissue sections of 240umx150um in area size (CamK2a-tTA/CamK2-Cre/Ai93, M, age P36). Dataset 1 size was defined based on the target of 1mm^3 volume, which is about the size for tracing axon connections between neurons across multiple cortical regions, and thus provide the more complete connectome at synaptic level. Dataset 2 and 3 are test datasets at different milestones of imaging pipeline buildup.
Data exclusions	No data were excluded. All the images were processed by automated stitching and alignment pipeline.
Replication	The is the state-of-the-art methodology for generation of large-scale neuroanatomical dataset. In addition to the 1mm^3 dataset mentioned in the manuscript (done by Dec 2018), we have imaged another two mm^3 volumes using the EM pipeline so far. One was 1mm^3 volume of a mouse V1 cortex, completed in 2019. The other one was 1mm^3 human tissue, completed in 2020.
Randomization	Randomization is not relevant to this study because all the images were automatically analyzed in the post imaging processing loop (Image QC, alignment and stitching).
Blinding	Blinding is not relevant to this study because all the image data were automatically analyzed in the post imaging processing loop (Image QC, alignment and stitching).

Reporting for specific materials, systems and methods

We require information from authors about some types of materials, experimental systems and methods used in many studies. Here, indicate whether each material, system or method listed is relevant to your study. If you are not sure if a list item applies to your research, read the appropriate section before selecting a response.

Materials & experimental systems

n/a	Involved in the study	
☒	☐	Antibodies
☒	☐	Eukaryotic cell lines
☒	☐	Palaeontology
☐	☒	Animals and other organisms
☐	☒	Human research participants
☒	☐	Clinical data

Methods

n/a	Involved in the study	
☒	☐	ChIP-seq
☒	☐	Flow cytometry
☒	☐	MRI-based neuroimaging

Animals and other organisms

Policy information about studies involving animals; ARRIVE guidelines recommended for reporting animal research

Laboratory animals	The lab animals used this study were mice, provided by Baylor College of Medicine. The mice went through 2P calcium imaging at BCM and were perfused at Allen Institute. All mice were housed in individually ventilated cages, 20-26 C, 30-70% Relative Humidity, with a 12-hour light/dark cycle.<br>Mouse (Slc-Cre/GCaMP6s, M, age P60)<br>Mouse (CamKII-tTA/tetO-GCaMP6s, M, age P49)<br>Mouse (CamK2a-tTA/CamK2-Cre/Ai93, M, age P36)
Wild animals	The study did not involve wild animal.
Field-collected samples	The study did not involve samples collected from field.
Ethics oversight	Approved by Allen Institute IACUC

Note that full information on the approval of the study protocol must also be provided in the manuscript.

Human research participants

Policy information about studies involving human research participants

Population characteristics	Tissue donors used in this study:<br>47 years old, male, neurosurgical donor, blood clot (Image data are displayed in Supplementary Movie 3.)
Recruitment	Neurosurgical specimens: Tissue procurement from neurosurgical donors was performed outside of supervision of the Allen Institute at local hospitals, and tissue was provided to the Allen Institute under the authority of the IRB of each participating hospital. A hospital-appointed case coordinator obtained informed consent from donors prior to surgery. To the best of our knowledge, the selection of participants does not represent of any bias in our data as our manuscript presents our imaging pipeline.
Ethics oversight	Tissue procurement from neurosurgical donors was performed outside of the supervision of the Allen Institute at local hospitals, and tissue was provided to the Allen Institute under the authority of the institutional review board of each participating hospital. A hospital-appointed case coordinator obtained informed consent from donors before surgery. Tissue specimens were de-identified before receipt by Allen Institute personnel. The specimens collected for this study were apparently non-pathological tissues removed during the normal course of surgery to access underlying pathological tissues. Tissue specimens collected were determined to be non-essential for diagnostic purposes by medical staff and would have otherwise been discarded.

Note that full information on the approval of the study protocol must also be provided in the manuscript.
